# Considerations in Understanding Vaccine Effectiveness

**DOI:** 10.3390/vaccines11010020

**Published:** 2022-12-22

**Authors:** Chin Shern Lau, Tar Choon Aw

**Affiliations:** 1 Department of Laboratory Medicine, Changi General Hospital, 2 Simei Street 3, Singapore 529889, Singapore; 2 Department of Medicine, National University of Singapore, Singapore 117599, Singapore; 3 Academic Pathology Program, Duke-NUS Medical School, Singapore 169857, Singapore

**Keywords:** vaccine effectiveness, prevalence, COVID-19, SARS-CoV-2

## Abstract

Although vaccine effectiveness reports are essential to assessing policies on SARS-CoV-2 vaccination, several factors can affect our interpretation of the results. These include the waning of antibodies, the prevailing viral variants at the time of the study, and COVID-19 disease prevalence in the population. Disease prevalence significantly impacts absolute risk reduction and could skew expected efficacy when increased disease prevalence inflates the absolute risk reduction in the face of a constant relative risk reduction. These factors must be considered in the interpretation of vaccine effectiveness to better understand how vaccines can improve disease prevention among the population. We highlight the impact of various factors on vaccine effectiveness.

## 1. Introduction

Vaccines have proven themselves as essential in the COVID-19 pandemic; for example, a Californian study estimated that SARS-CoV-2 vaccination averted up to 1.5 million COVID-19 cases and 19,430 deaths in their region [1]. Thus, reports of vaccine effectiveness (VE) are essential to guide public policy planning and informing physicians on when and how best to administer these vaccines. Indeed, vaccination has several spillover effects that benefit public health apart from its efficacy in preventing confirmed disease, such as a reduction of attack rates in unvaccinated individuals, a reduction of severe outcomes associated with the disease, and a net social and economic value [2]. Vaccine efficacy refers to the degree at which a vaccine prevents disease in an ideal/controlled setting (e.g., a clinical trial). However, VE refers to how a vaccine performs in real-world conditions. It is important to acknowledge that there are several factors that affect studies on the VE, including the host [3] and study/population factors [4]. In this commentary, we highlight some factors that can affect interpretations of VE, especially disease prevalence.

## 2. Post-Vaccination Antibody Kinetics and VE

Several studies have documented post-vaccination antibody kinetics [5,6,7,8]. However, although antibody levels and kinetics are essential, analyzing them in isolation has limitations as they are unlinked to clinical impact. One report [9] tried to obtain an immunological correlate of protection, and although they did find a good correlation between virus neutralization/antibody titers and efficacy (Spearman’s rank correlation coefficient of 0.79/0.93), it was still difficult to ascertain a definitive protective antibody titer. Furthermore, antibody titers steadily decrease 3–6 months after either the second or booster doses [5,8,10,11], corresponding with a decline in VE (especially in older individuals) [12].

Despite these declining trends, vaccination still has proven protective effects. One study [11] showed that even though post-booster memory B cell responses declined after four months, it was still a higher response compared to pre-booster levels. Despite waning effectiveness, even ≥5 months after 3 doses of any mRNA vaccine, VE was still 31% for emergency department encounters, and for hospitalizations VE was 36% ≥4 months post-booster during the Omicron wave [13]. In a recent report [14], booster vaccine doses also reduced transmission rates in prisons during the Omicron wave. Even after 6 months post-vaccination, the protection against severe disease still remained high, with one meta-analysis [15] finding that 81% of VE estimates against severe COVID-19 remained >70% six months after final vaccination. Thus, vaccination is still essential to reduce clinical disease and transmission, but it is also important to plot VE trends over time to determine the optimal timing for administering boosters, especially in older individuals.

## 3. Environmental Factors That Can Impact VE

The prevailing SARS-CoV-2 variant during the study can also significantly affect the reported VE at that point in time. When the Delta variant became predominant (from early to late 2021), overall, VE in preventing breakthrough infections was reduced from 90% to 78–81% [16]. When we experienced the Omicron wave from late 2021 to early 2022, the BNT162b2 vaccine showed reduced effectiveness in preventing symptomatic SARS-CoV-2 infection compared to during the Delta wave period [17], with recommendations for a booster vaccination to be given to prevent Omicron-related hospitalization/illness [18,19]. With the advent of newer variants (e.g., BA.4, BA.5, XBB) with enhanced immune evasion [20,21], VE against hospitalization was further compromised [22], with calls for additional doses of bivalent vaccine to improve protection [23,24]. Furthermore, the definition of VE has varied from study to study. Some studies report VE in terms of SARS-CoV-2 transmission [25,26], while others report VE in terms of prevention of hospitalization/ICU admissions [23,27,28]. Thus, it is important for VE studies to specify what their VE goal is.

Other environmental factors can also impact VE, for example, the compliance with non-pharmaceutical interventions by the populations to prevent disease. Although it is difficult to quantify the exact relationship between non-pharmaceutical interventions and VE, some mathematical models [29] predict that the combined effect of both has resulted in a 53% reduction in reproduction numbers, but vaccinations and non-pharmaceutical interventions alone had a 38% and 35% reduction, respectively. Another set of factors that can confound VE results are host factors. Age is well known to affect VE, with some studies reporting a maximum VE against infection of 83% vs. 76% between 18–44- and 45–64-year-olds [30]. Furthermore, certain drugs, such as immunosuppressants, can reduce the effectiveness of vaccines [31]. In addition, commonly available drugs such as ACE inhibitors and Angiotensin Receptor Blockers can confound VE studies by lowering the risk of mortality in COVID-19-infected hypertensive patients [32].

Thus, in any VE study, although there are some factors that can be controlled, it is important to acknowledge that there are several others that cannot, and these can influence the interpretation of VE.

## 4. Relative vs. Absolute Risk Reduction

The differences between relative risk reduction (RRR) and absolute risk reduction (ARR) are poorly understood by many doctors and patients. In vaccine efficacy trials, we are comparing the event rate (the proportion of individuals who present COVID-19) in the vaccinated group versus the unvaccinated group. The difference in event rates between these two groups expressed as a proportion of the event rate in the unvaccinated group is the RRR [(Cases in the placebo group − cases in the treatment group)/Cases in the placebo group], while the arithmetic difference in the event rates between the two groups is the ARR [(Cases in the placebo group/Total population of the placebo group) − (Cases in the treatment group/Total population of the treatment group)]. From the ARR, we can calculate the number needed to treat (NNT) as 1/ARR. The NNT would represent the number of people that need to be vaccinated to prevent one SARS-CoV-2 infection.

Thus, when we examine the original BNT162b2 vaccination efficacy study report [33], there were 162 cases in the placebo group (*n* = 18,325) and 8 cases in the vaccinated group (*n* = 18,198), which resulted in the RRR of around 95.1% [(162 − 8)/162], the often-cited VE of this vaccine. However, the ARR is surprisingly low at 0.84% [(162/18,325) − (8/18,198)], with a NNT of 119.0 (1/0.0084). Similarly, for the Moderna mRNA-1273 vaccine [34], the VE was reported as 94% [(185 − 11)/185], but the ARR was only 1.23% [(185/14,073) − (11/14,134)], with an improved NNT of 81.3 (1/0.0123) compared to the BNT162b2, making it the preferred vaccine in certain regions. Thus, both the RRR and the ARR (with the NNT) need to be examined in order to fully interpret VE studies.

## 5. The Effect of Prevalence on ARR

However, the prevalence of disease (total number of affected subjects/total population) has a significant impact on the ARR. For example, the prevalence of COVID-19 in the BNT162b2 study [33] is calculated as 0.47% [(162 + 8)/(18,325 + 18,198)]. The ARR changes greatly when prevalence is increased (while keeping the RRR fixed) (see Table 1).

We can also observe that the NNT decreases with increasing prevalence, indicating that the vaccine is most useful when the disease prevalence is increasing. In a similar fashion, if we increase the prevalence of disease in the mRNA-1273 study [34] (see Table 2):

As such, it is essential for physicians to also note the disease prevalence before deciding whether a vaccine is more effective or not based on the ARR as well as the NNT.

## 6. Conclusions

In summary, the amount of time since the last vaccination, the prevalence of the disease during the time of the study, and possible environmental/host factors need to be considered when interpreting VE studies. It is also important to remember that in studies, vaccine efficacy refers to the performance under ideal/controlled settings (e.g., a clinical trial), whereas VE is reported from real world settings. Only then would a more complete picture be obtained of how effective a vaccine is in our own populations during a certain period where a specific variant is predominant.

## Figures and Tables

**Table 1:** Effects of increasing prevalence of disease affecting ARR with a constant RRR in the BNT162b2 study (total population: 36,523) [33].

Prevalence	Total Cases	Vaccine Group (*n* = 18,198)		Placebo Group (*n* = 18,325)		Relative Risk Reduction	Absolute Risk Reduction	Number Needed to Treat
		Cases	Risk	Cases	Risk			
0.47%	170	8	0.04%	162	0.88%	95%	0.84%	119
1.00%	365	16	0.09%	349	1.90%	95%	1.81%	55.2
5.00%	1826	46	0.25%	1780	9.71%	95%	9.46%	10.6
10.00%	3652	160	0.88%	3492	19.06%	95%	18.18%	5.5

**Table 2:** Effects of increasing prevalence of disease affecting ARR with a constant RRR in the mRNA-1273 study (total population: 28,207) [34].

Prevalence	Total Cases	Vaccine Group (*n* = 14,134)		Placebo Group (*n* = 14,073)		Relative Risk Reduction	Absolute Risk Reduction	Number Needed to Treat
		Cases	Risk	Cases	Risk			
0.69	196	11	0.08%	185	1.31	94%	1.23%	81.3
1.00%	282	16	0.11%	266	1.89%	94%	1.78%	56.2
5.00%	1410	80	0.57%	1330	9.45%	94%	8.88%	11.3
10.00%	2821	165	1.17%	2656	18.87%	94%	17.70%	5.7

## Data Availability

All related data and methods are presented in this paper. Additional inquiries should be addressed to the corresponding author.

## Abbreviations

VE: Vaccine effectiveness; RRR: Relative Risk Reduction; ARR: Absolute Risk Reduction; NNT: Number Needed to Treat.
